# Neuromuscular electrical stimulation of the thighs in cardiac patients with implantable cardioverter defibrillators

**DOI:** 10.1007/s00508-016-1045-2

**Published:** 2016-07-25

**Authors:** Fadime Cenik, Dieter Schoberwalter, Mohammad Keilani, Bruno Maehr, Michael Wolzt, Maximilian Marhold, Richard Crevenna

**Affiliations:** 1 Department of Physical Medicine and Rehabilitation, Medical University of Vienna, Waehringer Guertel 18–20, 1090 Vienna, Austria; 2 Department of Cardiology, Hanusch-Krankenhaus, Vienna, Austria; 3 Therapiezentrum Rosalienhof, Versicherungsanstalt öffentlich Bediensteter, Bad Tatzmannsdorf, Austria; 4 Department of Clinical Pharmacology, Medical University of Vienna, Vienna, Austria; 5 Department of Internal Medicine I/Oncology, Medical University of Vienna, Vienna, Austria

**Keywords:** Implantable cardioverter defibrillator, Neuromuscular electrical stimulation, Heart failure, Risk, Electromagnetic interference

## Abstract

**Background:**

The aim of this systematic review was to update scientific knowledge concerning the safety of neuromuscular electrical stimulation (NMES) to increase exercise capacity and prevent cardiac cachexia in patients with implantable cardioverter defibrillators (ICDs).

**Methods:**

A systematic review including the electronic databases PubMed, MEDLINE, and SCOPUS was conducted for the time period from 1966 to March 31, 2016.

**Results:**

Only four articles fulfilled the inclusion criteria (three original articles/safety studies and one case report). The three (safety) studies used NMES to increase muscle strength and/or endurance capacity of the thighs. NMES did not show electromagnetic interference (EMI) with ICD function. EMI was described in a case report of 2 patients with subpectoral ICDs and application of NMES on abdominal muscles.

**Conclusion:**

This review indicates that NMES may be applied in cardiac ICD patients if 1) individual risks (e. g., pacing dependency, acute heart failure, unstable angina, ventricular arrhythmic episode in the last 3 months) are excluded by performing a safety check before starting NMES treatment and 2) “passive” exercise using NMES is performed only for thighs and gluteal muscles in 3) compliant ICD patients (especially for home-based NMES) and 4) the treatment is regularly supervised by a physician and the device is examined after the first use of NMES to exclude EMI. Nevertheless, further studies including larger sample sizes are necessary to exclude any risk when NMES is used in this patient group.

## Introduction

Typical symptoms of severe congestive chronic heart failure (CHF) are dyspnea, edema, weakness, and reduced functional capacity of skeletal muscles, which can result in cardiac cachexia [1–4]. Cardiac cachexia is a serious complication of CHF with a high morbidity and mortality, and is characterized by significant weight loss and muscle wasting [1, 2]. CHF-related muscle wasting is the result of an ongoing imbalance in the activation of anabolic and catabolic pathways [3, 5, 6]. This imbalance is caused by a series of immunologic, metabolic, and neuro-hormonal processes [2, 6, 7]. Efficient multidisciplinary care seems to be pivotal for disease management in this patient group [8].

Muscular strength of the thighs has been shown to be a predictor of long-term survival in CHF [1, 9, 10]. Patients with advanced CHF (New York Heart Association, NYHA, class IV) are generally excluded from active exercise to maintain muscle mass and functional capacity of skeletal muscle, namely endurance capacity and muscular strength [1, 10]. The physical treatment modality of neuromuscular electrical stimulation (NMES)—evoking muscle contractions by using electrical stimulation—is an established method to prevent atrophy of skeletal muscles [11–13]. It can be seen as an alternative option to active endurance and strength exercise for patients who are not allowed (contraindication) or unable (immobilization) to perform active exercise [14–17]. NMES leads to improvements in endurance capacity, muscular strength, and cross-sectional area of thigh muscles in patients with CHF, and has therefore been shown to be an effective option for “passive” exercise in these patients [17–19]. NMES has been described to effectively substitute, promote, or complement active exercise and increase adherence to rehabilitation protocols, especially for end-stage cardiac patients [17, 20]. Nevertheless, despite its benefits for exercise capacity and quality of life, NMES is currently underutilized in patients with CHF [21].

However, about 20 percent of CHF patients carry active electronic implants such as implantable cardioverter defibrillators (ICDs). Therefore, there is an existing and remaining concern of whether NMES can be applied in these patients, due to fear of electromagnetic interference (EMI). EMI can cause oversensing and lead to inappropriate therapies in ICD patients, resulting in arrhythmias and/or painful shocks [22–24]. These patients are generally advised to avoid exposure to electrical currents and electromagnetic sources, even though ICDs seem to be less sensitive to interference than pacemakers [25].

Pilot safety studies performed at the beginning of this century, designed to test the safety of NMES in patients with bipolar sensing by ICD lead systems, have shown that NMES treatment of knee extensor and flexor muscles seems to be safe and feasible in patients with bipolar sensing ICDs, providing that an individual risk is excluded before application [26, 27]. Nevertheless, several reports exist of inappropriate therapies/shocks due to interactions with electronic devices or electrical stimulation therapy leading to EMI [28–30].

This review aims to summarize and update knowledge from the scientific literature concerning NMES of thighs in ICD patients.

## Methods

A systematic review of the existing scientific literature was performed, including the electronic databases PubMed, MEDLINE, and SCOPUS. Electronic searches were conducted for the time period from 1966 to March 31, 2016. Trials with the keywords “Implanted Cardioverter Defibrillator”, “ICD”, “Neuromuscular stimulation”, “NMES”, “Functional electrical stimulation”, “FES”, and “Functional muscular stimulation” were extracted and considered for inclusion in the review. Furthermore, the combinations “Physical therapy and ICD and EMI” and “Physical therapy and ICD and Interference”, were also checked for inclusion in the review.

Studies were eligible for inclusion if they met the following criteria: original articles/safety studies, pilot studies, case reports, and reviews concerning the topic NMES in ICD patients were included. The restriction placed on language was that only English and German articles were included. The systematic literature search was performed separately by two independent researchers. Integration of their individual findings was supervised by two senior researchers [31, 32]. Author searches for key experts in the field were conducted for additional relevant articles. Furthermore, reference lists of each relevant publication were searched for additional information.

## Results

**Fig. 1:**
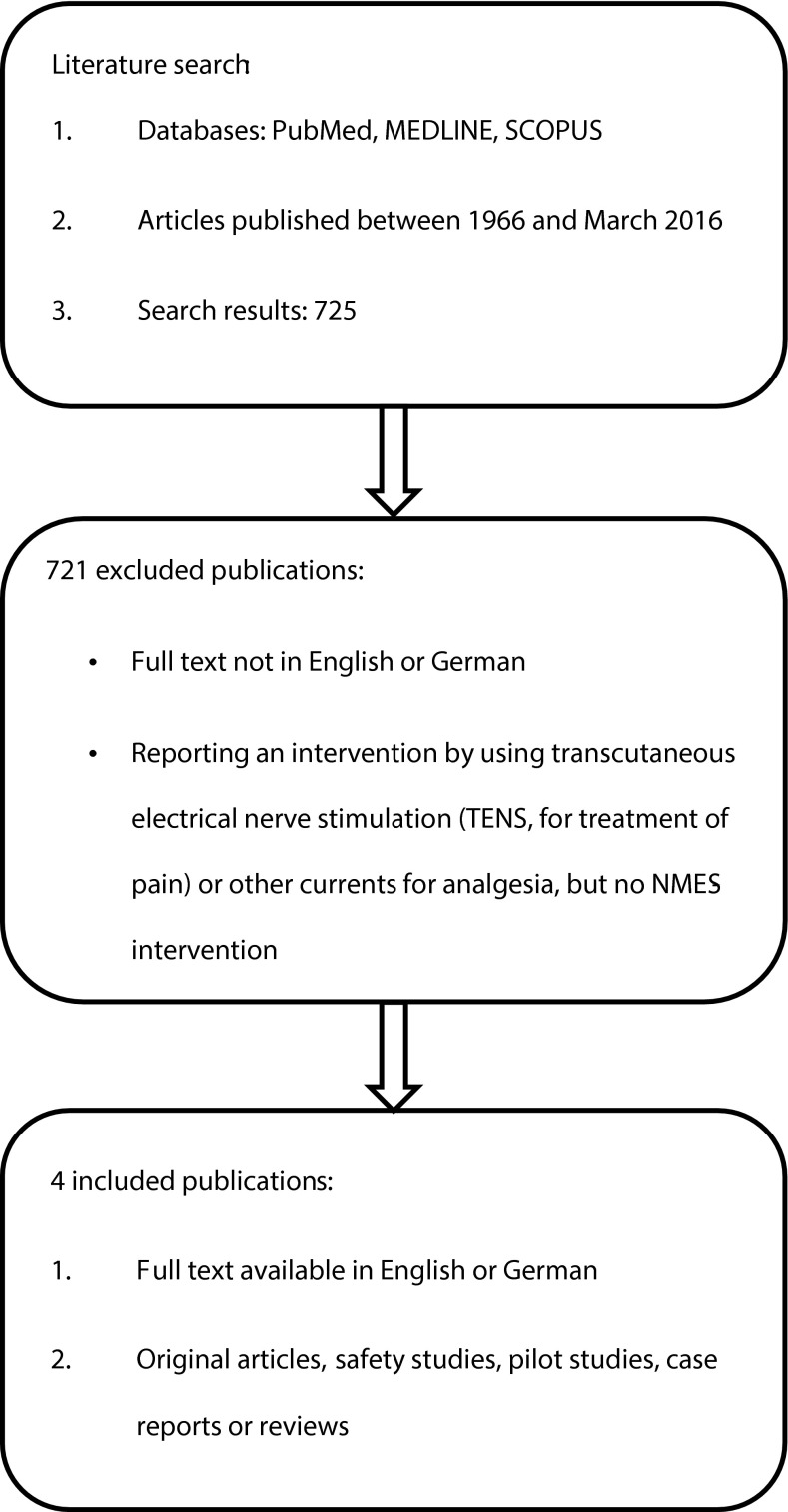
Flowchart of the systematic literature research and the selection process

Using the described search strategy, a total of 725 publications were found and screened for eligibility by title and abstract. 721 were rejected as non-includable or duplicates, and four studies were selected for full-text analysis (Fig. 1).

Of these, four fulfilled the inclusion criteria of being:

- Original articles (full text available in English or German),
- Safety studies, pilot studies, case reports, or reviews.

Studies were excluded because of the following criteria:

- Full text not available in English or German,
- Reporting an intervention using transcutaneous electrical nerve stimulation (TENS, for treatment of pain) or other currents for analgesia, but no NMES.

### Case report

Wayar et al. reported on two male patients with pectoral implanted ICDs (Ventak Mini III® and Ventak AV III DR®) and neuromuscular stimulation of abdominal muscles [33]. In both patients, NMES using commercial units (5.0–11.0 V, 7.3–10 mA, 55–75 Hz/biphasic) provoked EMI, resulting in ICD discharge due to misinterpreting electrical signals as cardiac signals in the ventricular fibrillation (VF) zone (Table 1; [33]).

**Table 1:** Summary of publications concerning neuromuscular electrical stimulation in cardiac patients with implantable cardioverter defibrillators

Author	Study design	Implanted device	Type/location of stimulation	Stimulation parameters	Results
Kamiya et al. 2016 [34]	Safety (pilot) study, (*n* = 27)	Left pectoral implanted ICD with dual-chamber lead system: – Medtronic Concerto® – 5x Medtronic Consulta® – 19x Medtronic Protecta® – 2x Boston Scientific Cognis 100-D®	NMES of quadriceps and gastrocnemius muscles	50 Hz, biphasic, 20 min, burst, 2.5 kHz, 5 s stim. +5 s interval, highest tolerable intensity (thigh: 25–60 mA; calf: 15–40 mA)	No EMI in all patients
Crevenna et al. 2004 [26]	Safety study (*n* = 6)	Subpectoral implanted ICD: – Medtronic 7223 CX® – Medtronic 7231® – Medtronic 7275® – Intermedics 101-10® – Guidant 1900® – Ventritex V‑190HV3®	NMES of thigh muscles	–4 patients: 63.3 Hz, 3.5 s on +4.5 s off, biphasic, 55–100 mA –2 patients: 15 Hz, 2 s on +4 s off, biphasic, 500 ms pulse width	No EMI in all patients
Crevenna et al. 2003 [27]	Safety study (*n* = 8)	Subpectoral ICDs: – ELA 9201® – St. Jude V‑230 HV® – Medtronic 7229 CX® – Guidant 1850® – Medtronic 7223 CX® – Guidant 1831® – Ventritex Contour MD® – ELA Defender IV®	– IG 50, FM, HF TENS, LF TENS of trapezoid muscle – E200, aS, aS1, FIB of thigh muscles	– IG50: dir. cur., 128 mA, 200 Hz, 400 μs stim. + serial duration of 50 ms – FM: 3.33–33.3 Hz, 400 μs alternated by tetanizing impulse effects – HF-TENS: 100 Hz, 200 μs stim. – LF-TENS: 2 Hz, 200 μs stim. – E200: 200 ms rise +270 ms pulse dur., 0.44 Hz; – aS: 400 μs pulse dur. +6.5 s thresh. dur., 66.7 Hz – aS1: 400 μs pulse dur. +3.6 s thresh. dur., 66.7 Hz – FIB: 60 ms impulse with an interval of 200 ms; – Home treatment devices: – biphasic, 500 μs impulse, 15 Hz, 2 s pulse dur. – biphasic, 250 μs impulse, 8/15/30 and 50 Hz, 1 s rise time, steady impulse over 8 s, 1 s fall time	EMI caused by – FM in 1 patient – LF TENS in 2 patients – FIB in 2 patients *Neck stimulation*: FM stimulation in 1 patient/LF TENS in 2 patients: EMI of ventricular sensing/atrial sensing. *Thigh stimulation*: FIB: EMI in 2 patients and intermittent ventricular undersensing due to postsense *EMI did not fulfill ICD detection criteria of a tachyarrhythmic ventricular episode in any of the subjects under study blanking in 1 of these participants.* *Home therapy (NMES) of thighs*: no EMI
Wayar et al. 2003 [33]	Case report (*n* = 2)	1st patient: pectoral ICD (Ventak® Mini III [Guidant Inc., St. Paul, Minnesota, USA])	NMES of abdominal muscles	55–75 Hz, biphasic, 7.3–10 mA, 5–11 V	EMI in both patients
		2nd patient: pectoral ICD (Ventak® AV [Guidant Inc., St. Paul, Minnesota, USA])			

*ICD* implantable cardioverter defibrillator, *NMES* neuromuscular electrical stimulation, *stim* stimulation, *EMI* electromagnetic interference, *IG50* Impulse galvanization, *dir. cur.* direct current, *FM* frequency modulation, *HF-TENS* high frequency transcutaneous electrical nerve stimulation, *LF-TENS* low frequency transcutaneous electrical nerve stimulation, *FIB* mimics tachycardia, *thresh. dur.* threshold duration, *Stiwell 1200 (Med-El, Innsbruck, Austria) and Compex 2 (Compex SA, Ecublens, Switzerland)* home devices

### Safety studies

Crevenna et al. performed the first safety study [27]. Eight patients had subpectorally implanted ICDs with transvenous bipolar sensing leads and received different types (different current forms) of electrical stimulation of trapezoid and thigh muscles, namely impulse galvanization (IG50); frequency modulation (FM); high-frequency transcutaneous electrical nerve stimulation (HF-TENS) and low-frequency transcutaneous electrical nerve stimulation (LF-TENS) of trapezoid muscles; “E200” (impulses of a rise duration of 200 ms and pulse duration of 270 ms at a frequency of 0.44 Hz); “aS” (pulse duration of 400 µs with a threshold duration of approximately 6.5 s at a frequency of 66.7 Hz); “aS1” (pulse duration of 400 µs and a threshold duration of 3.6 Hz at a frequency of 66.7 Hz); and a (specially programmed) electrical current (“FIB”) comprising triangular impulses over 60 ms with an interval of 200 ms, mimicking an episode of tachycardia in the ICD detection zone. Furthermore, two different commercially available devices for home therapy for increasing endurance capacity (Stiwell 1200 (Med-El, Innsbruck, Austria) and Compex 2 [Compex SA, Ecublens, Switzerland]) were tested.

The results showed that electrical stimulation was well tolerated in all participants and no clinical side effects were reported. No interference with ventricular sensing during electrical stimulation was seen in 5 of 8 patients. No EMI was caused upon stimulating the thigh musculature using home therapy devices or during stimulation protocols “E200”, “aS”, or “aS1”. Mimicking a tachycardia by using “FIB” to thigh muscles was enforced in 2 patients. EMI of ventricular sensing was also caused by stimulating trapezoid muscles during FM in 1 patient and during LF-TENS in 2 patients. The authors advise investigating EMI before starting NMES in patients with ICDs (Table 1; [27]).

In another study, Crevenna et al. assessed the safety of long-term NMES in patients with ICDs [26]. Six patients with subpectoral ICDs were exposed to long-term NMES of thigh muscles. Four inpatients received NMES to increase muscle strength (biphasic symmetric pulses with pulse duration of ±400 ms at a frequency of 63.3 Hz; 3.5 s on, 4.5 s off). Two outpatients performed NMES as home treatment to increase endurance capacity (biphasic symmetric pulses of 500-ms pulse width at a frequency of 15 Hz; 2 s on and 4 s off). During NMES, all participants together received 14,139,799 biphasic electrical pulses and 412,425 on-phases without adverse events. No abnormalities of ICD function were recorded after the stimulation period in any patients. In a safety procedure, NMES was applied under supervised conditions to evaluate the individual risk. Symptom-limited NMES was performed and the ICD was interrogated online for potential occurrences of EMI. The results of this indicated that long-term NMES of thigh muscles seems to be safe in patients with ICDs, providing that an individual risk was excluded prior to start of NMES therapy (Table 1; [26]).

Kamiya et al. recently performed a safety study with NMES in 27 patients with left pectoral ICDs [34]. Medium-frequent NMES of knee flexors and extensors with alternating sinusoidal current (2.5 kHz) for 20 minutes in bursts with a carrier frequency of 50 Hz and impulse trains for 5 s and pauses for 5 s was used at individual highest tolerable intensities. In this case, NMES of thigh muscles was applied to the participants without any occurrence of EMI. Additionally, EMI between ICDs and NMES application was not observed. The authors therefore concluded that NMES of thigh and calf musculature can be safely applied to patients with an ICD (Table 1; [34]).

## Discussion

Muscular strength of thighs has been shown to be a predictor of long-term survival in CHF [1, 9, 10]. As Parissis et al. have summarized, functional electrical stimulation of thighs and calf muscles is the only alternative mode of exercise in patients with CHF [18].

Literature about NMES in patients with ICDs is very rare. The reason for this seems to be that ICDs are generally seen as a contraindication for the use of NMES by the community. Nevertheless, NMES has been described to be an effective and safe treatment option for ICD patients provided certain safety precautions are considered [26, 27, 34]. In the scientific literature, there are only four articles describing the safety of NMES in patients with pectoral ICDs. Three of them showed the safety of NMES when applied to lower extremities in order to improve muscle strength and endurance capacity [26, 27, 34]. Crevenna et al. reported feasibility and safety of NMES in ICD patients if individual risks were checked prior to treatment initiation [26, 27].

In contrast, inappropriate electrode positioning for NMES can provoke disturbances of ICDs [33]. In one study, 2 patients autonomously applied the stimulation too close to the implantation site of the pectoral ICD, namely to the abdominal musculature, causing discharge of the ICD by signals in the VF zone [33]. Nevertheless, at that time, ICD programming had usually been very strict, e. g., short detection of VF and shock delivery at the first stage in the VF zone. Nowadays, prolonged detection programming and shock delivery only if antitachycardia pacing etc. fail may prevent many inappropriate shocks. Therefore, it might be doubtful that modern ICDs can be disturbed by NMES in the abdominal region. Nevertheless, further studies are needed to answer this question.

Unfortunately, the terms TENS (for pain treatment) and NMES are not clearly distinguished by all publishing authors. TENS is a non-pharmacologic treatment for pain relief, applied in the form of low-frequency (<12 Hz) or high-frequency TENS (50–100 Hz). Some case reports present discharge of pectoral or abdominal ICDs in patients during use of TENS for pain treatment in the lumbar, trapezoidal, and abdominal musculature, or chest or midback area [27–30, 35]. In another case report, TENS of the sacral region provoked an ICD discharge, although the electrodes were 12 inches away from the ICD pulse generator [36]. In an exploratory study, 30 patients with implanted defibrillators (ICDs) underwent TENS treatment above the mammillae and the anterior superior iliac spine after programming the ICD to monitoring mode. Due to possible consequences of inexact sensing, the authors do not advise the application of TENS in patients with an ICD [37]. Pain management using electrotherapy, e. g., TENS, is substituted by pharmacological treatment and should be avoided in patients with ICDs.

Options to improve muscle strength and endurance capacity in patients with end-stage heart failure are rather limited. In most cases, patients with CHF are discouraged from performing active exercise to maintain muscle mass and functional capacity of skeletal muscle, namely endurance capacity and muscular strength [1, 11]. In Austria, NMES treatment of patients suffering from chronic diseases has a long tradition [17, 20, 26, 27, 38–42]. Depending on the exercise goal, different commercially available therapy devices are used with direct or alternating current (biphasic) between 8–63.3 Hz, and, according to this, adjustable pulse durations and session length (usually 20–30 min).

This systematic review of existing scientific literature indicates that NMES treatment of thigh muscles seems to be safe and feasible in CHF patients with bipolar ICDs. Nevertheless, risk analyses to detect harmful events require a large sample size in order to arrive at valid predictions for the community, and such high powered and large safety studies are yet to be realized. We therefore conclude that NMES can be performed in cardiac ICD patients if 1) individual risks (e. g., pacing dependency, acute heart failure, unstable angina, ventricular arrhythmic episode in the last 3 months) are excluded by performing a safety check before starting NMES treatment and 2) “passive” exercise using NMES is performed only for thighs and gluteal muscles in 3) compliant ICD patients (especially for home-based NMES) and 4) the treatment is regularly supervised by a physician and the device is examined after the first use of NMES to exclude EMI. Nevertheless, further studies including larger sample sizes are necessary to exclude any risk when NMES is used in this patient group.
